# Description of *Lentistivalius philippinensis*, a new species of flea (Siphonaptera, Pygiosyllomorpha, Stivaliidae), and new records of Ascodipterinae (Streblidae) on bats and other small mammals from Luzon, The Philippines

**DOI:** 10.3897/zookeys.260.3971

**Published:** 2013-01-18

**Authors:** Michael W. Hastriter, Sarah E. Bush

**Affiliations:** 1 Monte L. Bean Life Science Museum, Brigham Young University, P.O. Box 20200, Provo, Utah 84602-0200, U.S.A.; 2 Department of Biology, University of Utah, Salt Lake City, Utah 84112, U.S.A.

**Keywords:** *Ascodipteron*, bat flies, fleas

## Abstract

During May 2009 and July 2011, we collected 357 mammals and examined each for ectoparasites. Among the ectoparasites collected, a new species of flea was discovered. This new species, *Lentistivalius philippinensis*, is described from the male sex only. Two males were recovered from two specimens of the soricid *Crocidura grayi* Dobson in Municipality Maria Aurora, Aurora Province, Luzon, Philippines. Additional fleas included *Thaumapsylla breviceps orientalis* Smit, *Thaumapsylla longiforceps* Traub, and *Ischnopsyllus indicus* Jordan. Although the latter species is common in Japan and documented in Guam (as well as mainland Southeast Asia) also on *Pipistrellus javanicus* (Gray), *Ischnopsyllus indicus* represents a new record in the Philippine Islands. The ascodipterinae (Streblidae) *Maabella stomalata* and *Ascodipteron speiserianum* Muir collected from *Rhinolophus inops* K. Andersen and *Rhinolophus subrufus* K. Andersen, respectively, also represent new host records. A key to the species of the flea genus *Lentistivalius* Traub is provided.

## Introduction

During May 2009 and July 2011, we collected 357 mammals representing 57 species from the Philippines and examined each for ectoparasites. All but one of these mammals were collected from the island of Luzon in the northern Philippines. One bat was collected on the island of Negros in the southern Philippines. Bat flies in the families Nycteribiidae and Streblidae (Diptera) were present on many of the bat specimens, including one unusual group of endosomic flies in the subfamily Ascodipterinae (Streblidae). In addition to the ascodipterons, several species of bat fleas and a new species of flea in the Siphonapteran suborder Pygiopsyllomorpha are reported in this study. Molecular and morphological analyses of nycteribiid and other non-endosomic streblid flies from bats will be reported in a separate paper.

## Materials and methods

**Figure 1.**
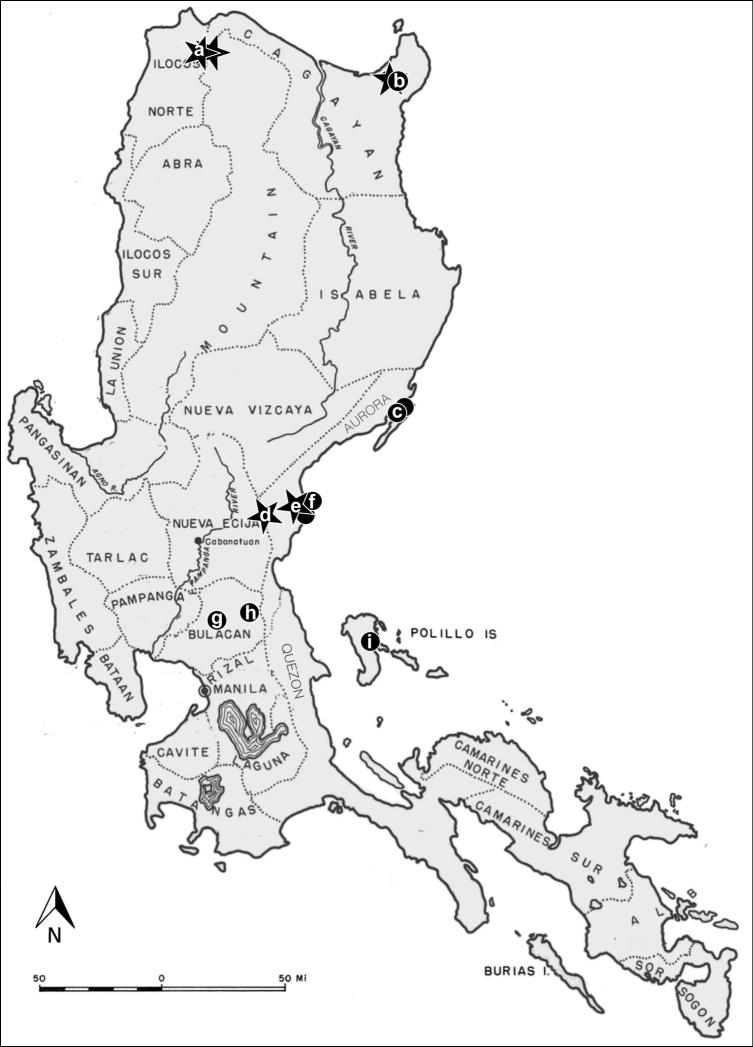
Sampling locations on the island of Luzon (n = number of mammals examined at each site). Stars indicate the location of ascodipterons and fleas discussed in this paper. **a** Mt. Pao (n=38) and Mt. Cabacan (n=38) **b** Mt. Cagua site 1 (n=62) and Mt. Cagua site 2 (n=32) **c** Casiguran (n = 16) **d** Maria Aurora (n=34) **e** Sitio Minoli (n=56) and nearby field site (n=24) **f** Zabali (n=39) **g** Tower site (n=14) **h** Angat (n=3); and **i** Burdeos (n=1). Photo courtesy of S. Villa.

Mammals and their ectoparasites were surveyed at 12 field sites on the island of Luzon (Fig. 1), and one bat was collected from the island of Negros. A map for the island of Negros is not included. Mammals were captured and euthanized according to guidelines of the American Society of Mammalogists (Gannon et al. 2007). Mist nets and harp traps were set in the forest and at, or near cave entrances to capture bats. Bats were processed for ectoparasites in accordance with Hastriter and Bush (2006). Terrestrial mammals were captured with Sherman traps or snap-traps. Each mammal was subjected to a thorough post-mortem visual examination: the face and ears were carefully searched and parasites were removed with forceps. In addition, the fur was systematically searched with the aid of a fine-toothed metal comb (LiceMeister^™^, National Pediculosis Association, Needham, MA). All ectoparasites recovered were preserved in 95% ethanol for later processing and identification in the laboratory. All associated hosts were prepared as museum specimens and were deposited in the Kansas Museum of Natural History (KUMNH), Lawrence, KS, U.S.A. Siphonaptera and Ascodipterinae were deposited in the Monte L. Bean Life Science Museum, Brigham Young University, Provo, UT, U.S.A.

## Results

### Diptera Streblidae: Ascodipterinae

#### Ascodipteron speiserianum

Muir, 1912

http://species-id.net/wiki/Ascodipteron_speiserianum

##### Material examined.

Philippines, Luzon Island, Aurora Province: Sitio Minoli, Municipality San Louis (15.680°N, 121.529°E), elev. 520m, *Rhinolophus subrufus* K. Andersen (JAE2961), 13 VI 2009, K. Dittmar (1dealate ♀ w/o caudal disc, P-2661).

##### Remarks.

Only one *Ascodipteron speiserianum* was collected from the 19 *Rhinolophus subrufus* specimens that were examined. *Ascodipteron speiserianum* was documented in the Philippines in Hastriter (2007) from Rizal Province, Luzon from a “bat”. The site of attachments of *Ascodipteron speiserianum* is commonly at base and behind ear pinna (less commonly on the body) on species of the bat genus *Miniopteru*s Bonaparte. Its presence on *Rhinolophus subrufus* represents a new host record.

#### Maabella stomalata

Hastriter & Bush, 2006

http://species-id.net/wiki/Maabella_stomalata

##### Material examined.

Philippines, Luzon Island, Cagayan Province: Mt. Cagua 2, Magrafil Barangay (18.236°N, 122.104°E), elev. 680m, *Rhinolophus inops* K. Anderson (JAC093), 20 VII 2011, S. Villa and S. Knutie, (1 dealate ♀ w/o caudal disc, P4631); same data except *Rhinolophus inops* (JAC094) (1dealate ♀ with caudal disc, P4632); same data except *Rhinolophus inops* (JAC096) (1 dealate ♀ w/o caudal disc and 1 dealate ♀ with caudal disc, P4640); and same data except *Rhinolophus inops* (JAC097) (1dealate ♀ with caudal disc, 2 dealate ♀♀ w/o caudal discs, P4636).

##### Remarks.

**Figures 2–3.**
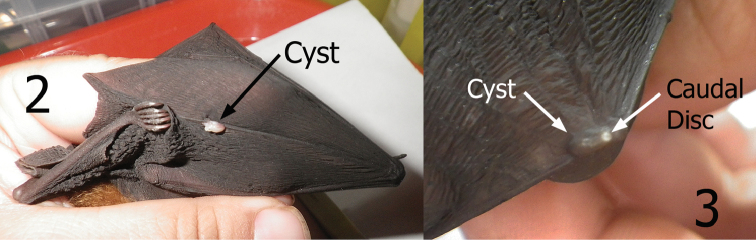
Cysts containing neosomes of *Maabella stomalata*. **2** Cyst located on dorsal surface directly over wing digit number five of *Rhinolophus* sp. (species undetermined) **3** Cyst located on dorsal surface directly over the radius-ulna/thumb joint of *Rhinolophus inops*. Arrow depicts caudal disc (spiracle breathing structure) protruding through the skin.

*Maabella* is a widespread monotypic genus. Hastriter and Bush (2006) described *Maabella stomalata* from China and Vietnam with records from *Rhinolophus affinis* Horsfield, *Rhinolophus macrotis* Blyth, and *Rhinolophus paradoxalophus* (Bourret). Subsequently Hastriter (2007) documented a *Maabella stomalata* in Borneo, Java, Moluccas, Malaysia, Myanmar, Papua New Guinea, Philippine Islands, and West Papua and cited the additional bat host species of *Rhinolophus acuminatus* Peters, *Rhinolophus euryotis* Temminck, *Rhinolophus megaphyllus* Gray, *Rhinolophus rufus* Eydoux and Gervais, *Hipposideros calcuratus* (Dobson), *Hipposideros cervinus* (Gould), and *Rousettus amplexicaudatus* (E. Geoffroy). Although our study does not expand the distribution of *Maabella*, *Rhinolophus inops* represents a new host record. Members of the bat family Rhinolophidae are the preferred hosts of *Maabella stomalata*. Its occurrence on *Rousettus amplexicaudatus* is probably an accidental association. The site of penetration of neosomes has commonly been found on the leading edge of the wing and over the joints of the front part of the wings (Hastriter, 2007). Our specimens were also found over wing bones and joints (Figs 2–3) with occasional specimens in the skin or “patagia” of the wings unassociated with bones. Locations over the bones/joints of the wing might be an adaptation of *Maabella* to prevent suffocation by the host’s skin from blocking the spiracles that protrude through the host’s thin skin via the caudal disc. The underlying wing bones are also more open to surface air when wings are folded during rest/sleep. Note in Figs 2 and 3 that the neosomes within the cysts lay horizontal to the surface. *Ascodipteron* species that occupy body tissues penetrate deeper and arrange themselves (*in situ*) perpendicular to the skin surface (not horizontal).

**Figures 4–7.**
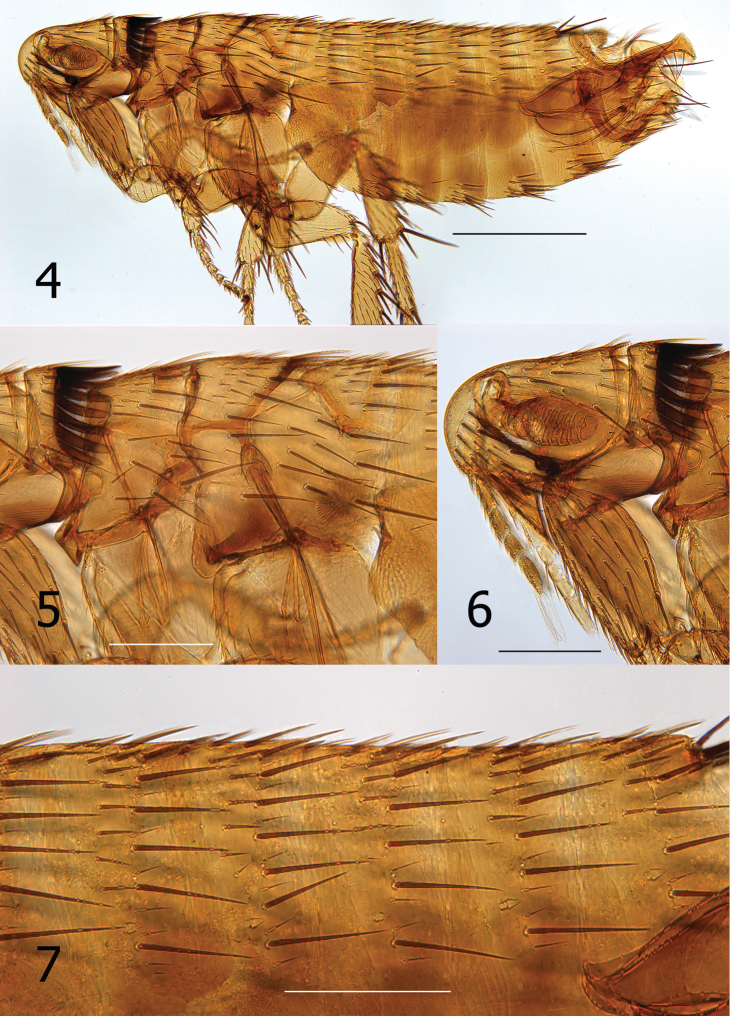
*Lentistivalius philippinensis* sp. n. (P2316) **4** Overview, male holotype **5** Thorax **6** Head, pronotum, forecoxa **7** Abdominal tergites. (Scale: Fig. **4** = 100 µ; Figs **5–7** = 200µ).

### Siphonaptera Ischnopsyllidae, Thaumapsyllinae

#### Thaumapsylla breviceps orientalis

Smit, 1954

http://species-id.net/wiki/Thaumapsylla_breviceps_orientalis

##### Material examined.

Philippines, Luzon Island, Aurora Province: Sitio Minoli, Municipality San Louis (15.680°N, 121.529°E), elev. 520m, *Eonycteris robusta* Miller (JAE2944), 12 VI 2009, K. Dittmar, (1♂, P2650); same except *Eonycteris spelaea* Dobson (JAE3021), 16 VI 2009 (1♀, P2766); same except *Eonycteris spelaea* (JAE3023) (1♀, P2770); same except *Rousettus amplexicaudatus* (JAE3027) (1♀, P2775); same except *Eonycteris spelaea* (JAE3040) (1♂, P2779); Luzon Island, Ilocos Norte Province, Adams village, Mt. Pao, (18.438°N, 120.878°E), elev. 750m, *Rousettus amplexicaudatus* (NCA049), 22 VI 2011, S. Villa, (2♀♀, P4196); Luzon Island, Ilocos Norte Province, Adams village, Mt. Cabacan, (18.449°N, 120.894°E), elev. 475m, *Rousettus amplexicaudatus* (NCA125), 30 VI 2011, S. Villa, (1♀, P4318); Luzon Island, Cagayan Province, Barangay Magrafil (closest city Gonzaga), Mt. Cagua, (18.219°N, 122.111°E), elev. 780m, *Rousettus amplexicaudatus* (JAC036), 11 VII 2011, S. Villa and S. Knutie, (1♂, P4496).

##### Remarks.

The two populations of *Thaumapsylla breviceps* are recognized (*Thaumapsylla breviceps breviceps* Rothschild, 1907 and *Thaumapsylla breviceps orientalis* Smit, 1954). The nominate species is found in southern portions of Africa and the other in the Oriental and Australasian regions. Beaucournu and Kock (1994) provide a distributions map of *Thaumapsylla breviceps orientalis*. There has been some controversy regarding the validity of *Thaumapsylla breviceps orientalis*. Smit (1954) erected *Thaumapsylla breviceps orientalis* after studying material from both geographical regions and reported only one distinguishing character in males: the presence of a convex margin along the apex of the basimere in the nominate subspecies vs. a slight concavity along the margin in the other subspecies. He found no differences in females from the respective regions and considered them indistinguishable. All three males examined in our study present a distinct concavity in the apex of the basimere. Females are identified based only on geographic distribution. Material reported by Hastriter and Bush (2010) as “*Thaumapsylla breviceps* Rothschild”, belong to *Thaumapsylla breviceps orientalis* Smit, 1954. These two subspecies parasitize fruit bats of the genus *Rousettus* in both regions. Although *Thaumapsylla breviceps orientalis* was found on four *Rousettus* and four *Eonycteris* bats, the prevalence of this flea was highest on *Rousettus* bats. Of 11 *Rousettus* specimens examined, 36% harbored this flea, whereas only 18% of the 22 specimens of *Eonycteris* were infested.

None of the three species on which *Thaumapsylla breviceps orientalis* occurred represent new host records.

#### Thaumapsylla longiforceps

Traub, 1951

http://species-id.net/wiki/Thaumapsylla_longiforceps

##### Material examined.

Philippines, Luzon Island, Ilocos Norte Province, Adams village, Mt. Pao, (18.438°N, 120.878°E), elev. 750m, *Eonycteris robusta* (NCA055), 23 VI 2011, S. Villa, (1♂, P4222); Luzon Island, Ilocos Norte Province, Adams village, Mt. Cabacan, (18.449°N, 120.894°E), elev. 475m, *Eonycteris robusta* (NCA081), 27 VI 2011, S. Villa, (1♀, P4253); same data except *Rousettus amplexicaudatus* (NCA125), 30 VII 2011, S. Villa, (1♀, P4318).

##### Remarks.

*Thaumapsylla longiforceps* is not as widespread in the Oriental region as *Thaumapsylla breviceps orientalis*. These two species may occur on the same host as we found a female of each flea species on host NCA125 (*Rousettus amplexicaudatus*). This species commonly occurs on pteropodid bats (fruit bats) but has also been documented on vespertilionid and rhinolophid bats.

### Ischnopsyllidae, Ischnopsyllinae

#### Ischnopsyllus (Hexactenopsylla) indicus

Jordan, 1931

http://species-id.net/wiki/Ischnopsyllus_indicus

##### Material examined.

Philippines, Negros Island, Mt. Bungal, Northern Negros Natural Park (10.674°N, 123.189°E), elev. 1200m, *Pipistrellus javanicus* (Gray) (JAE3252), 23 VII 2009, J. Esselstyn, (1♂).

##### Remarks.

*Ischnopsyllus indicus* has been documented in China, Taiwan, Vietnam, India, Guam, Sri Lanka, and Japan from a number of vespertilionid bat species; however, this is the first record of *Ischnopsyllus indicus* in the Philippines. Finding this flea in the Philippines is no surprise, since *Ischnopsyllus indicus* was documented in Guam by Jordan (1941), in Japan by Hopkins and Rothschild (1956), and is very common in Japan according to Sakaguti and Jameson (1962) on the same host species (*Pipistrellus javanicus*) on which we found this species. *Pipistrellus javanicus* is widely distributed in eastern Russia, China, south and central Japan, Southeast Asia through the Sunda Islands, and in the Philippines (Wilson and Reeder 2005). Several differences should be noted in our specimen and those illustrated in the original male description by Jordan (1941) and subsequently copied by Hopkins and Rothschild (1956). The telomere appears more oblique at its apex. There are a pair of flat ribbon-like, long curved setae at the apex (one pair on each side) of S-VIII. This does not appear illustrated as such by either Jordan or Hopkins and Rothschild. These ribbon-like setae are absent in all other species of the subgenus *Hexactenopsylla*. The illustrations of Sakaguti and Jameson (1962) more accurately depict the features of our single male from Luzon.

### Stivaliidae, Stivaliinae

#### Lentistivalius philippinensis

Hastriter & Bush sp. n.

urn:lsid:zoobank.org:act:5E6F547E-51A0-40C2-A292-DDEB2B2A2D12

http://species-id.net/wiki/Lentistivalius_philippinensis

Figs 4 [Fig F4] [Fig F5] –15

##### Type material.

Holotype male (P2316), Philippines, Luzon Island, Aurora Province: Camp 1, Municipality Maria Aurora (15.685°N, 121.343°E), elev. 507m, *Crocidura grayi* Dobson (JAE2825), 25 V 2009, K. Dittmar and V. Tkach; 1♂ paratype (P2211), same data except *Crocidura grayi* (JAE2785), 22 V 2009. Holotype deposited in the Carnegie Museum of Natural History, Pittsburgh, PA and male paratype in the Brigham Young University flea collection, Monte L. Bean Life Science Museum, Provo, UT.

##### Diagnosis.

**Figures 8–10. F4:**
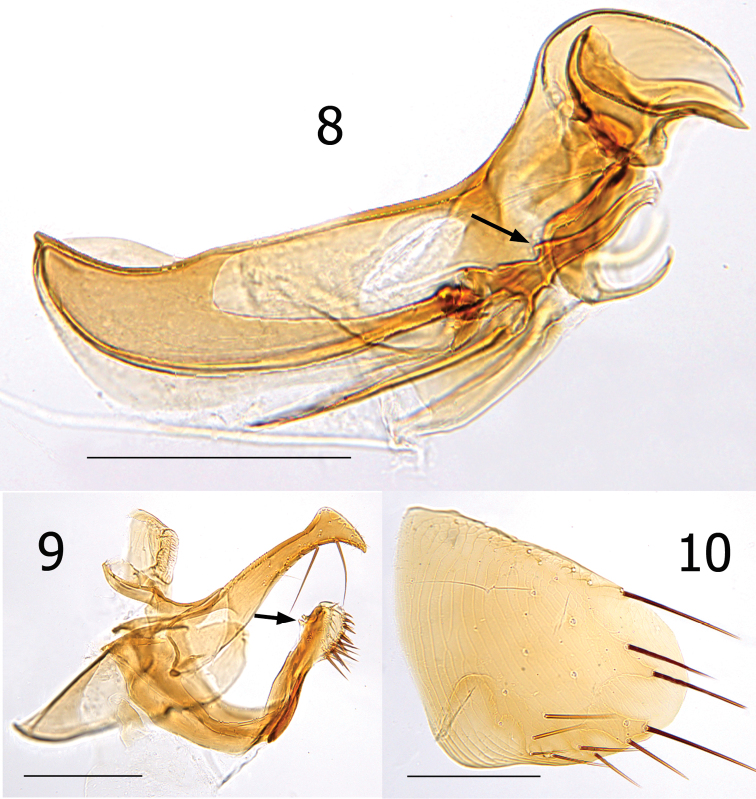
*Lentistivalius philippinensis* sp. n., male paratype (P2211). **8** Aedeagus **9** Tergum IX and Sternum IX **10** Sternum VIII. (Scale: 200µ).

Female unknown. Male easily distinguished from all species except *Lentistivalius aestivalis* by the presence of a prominent spur along dorsal margin at base of sclerotized inner tube (Fig. 8). Further distinguished from *Lentistivalius aestivalis* by the narrow width of the distal half of the crochet (Fig. 8); width does not exceed width of sclerotized inner tube in the new species whereas it does in *Lentistivalius aestivalis*. Other distinguishing features include the shapes of the distal arm of S-IX, crochet, and Ford’s sclerite (Figs 8–9).

##### Description.

Numbers of setae described indicate only one side unless otherwise stated. Head (Figs 4, 6). Frons smoothly rounded; punctate area extensive anterior to frontal row of six moderately heavy setae. Ocular and genal rows: three setae each. Seven supernumerary setae between frontal row and ocular row. Two labral setae. Four minute setae line ventral rim of antennal fossa anterior to eye. Maxillary palpus extends to mid coxa; labial palpus of five segments (excluding basal segment); apical segment longest. Labial palpus extending ¾ length of forecoxa. Darkly pigmented eye contiguous with genal margin; with ventral sinus. Post-antennal area with four rows setae (3, 4, 1, 5 + intercalaries). Numerous setulae along antennal fossa. Four lateral setae on scape; four short apical setae on pedicel. Clavus not extending beyond caudal margin of head. Thorax (Figs 4, 5). Pronotum with 18 ctenidia (both sides); each outside tooth much smaller than others. Longest ctenidia twice length pronotum, about equal vertical length pronotum; each tooth divergent, curved dorsally. Two rows setae; anterior row with two setae. Meso- and metanota each with three rows setae. Metanotum with single sharp hyaline spine at ventrocaudal margin. Prosternosome without notch for 1^st^ link-plate; not extended ventrally on ventral margin. Mesosternum reduced; extending ventrally between coxae as triangular projection. Mesepisternum with three setae; mesepimeron with six setae, single posterior seta largest. Pleural rod, bifurcate dorsally. Metasternum rounded; metepisternum with single large seta and single minute seta. Squamulum long, narrow. Pleural arch well developed; pleural ridge more robust dorsally. Well defined suture between lateral metanotal area and metepisternum. Metepimeron with three vertical rows setae (3, 4, 3), all below level pointed spiracular atrium; posterior setae longest. Legs (Figs 12–15). Forecoxa heavily adorned with setae. Anterior margins meso- and metacoxae with numerous setae along lower two thirds. Oblique lateral sulcus of mesocoxa complete. Two setae each guarding femorotibial joints of all three tibiae; outer short, spiniform, inner seta many times longer. Lateral surface of hind femur with coarse horizontal parallel sculpturing; mesal surface with broader vertical parallel sculpturing (perpendicular to longitudinal axis of femur). First tarsal segment of foreleg with unique set of three long setae along posterior margin. Tarsal segments 1-4 each leg progressively shorter (proximal to distal) than preceding segment. First tarsal segment hind leg nearly as long as segments 2–4. Dorsal margins all tibia with seven notches; setae per notch metatibia (2, 2, 1, 2, 2, 1, 3). Lateral surface metatibia covered with usual setae; none enlarged or shifted towards dorsal notches. Six lateral plantar bristles each distitarsus. Fifth segment of fore and mid distitarsi with four spiniform preapical plantar bristles; hind distitarsus with two small preapical plantar bristles. Three proximal lateral plantar bristles more robust than distal three pairs; third and fourth at same level, third inside and fourth outside. Unmodified Abdominal Segments (Figs 4, 7). Tergites I-VII with three rows setae; anterior row one to two setae. Terga II-V with single apical pigmented spinelet. Two antesensilial bristles; lateral twice length of mesal. Sternum II, three ventral setae; single small seta near rod-like fourth link-plate. Dorsocephalic margin S-II heavily sclerotized; incrassation at fourth link-plate. Sterna III-VII with four setae main row; 5–12 scattered setae preceding main rows. Modified Abdominal Segments (Figs 4, 8–10). Tergum VIII reduced, with two small dorsal setae; spiracle VIII large, equal to convex sensilium. Subsensilial sclerite present; bearing two setae. Sternum VIII largely covering T-IX, S-IX, and aedeagus; numerous setae on apical two thirds. Proximal arm of S-IX apically broad and blunt, fused with manubrium. Distal arm of S-IX strongly sclerotized along ventral margin; apex expanded, club-like. Club with small lateral patch of setulae; oblique line of eight setae (distal six fine, proximal two long, pigmented), ventroapical margin with six setae (distal four short, spiniform, proximal two long, all darkly pigmented). Lacking apical lobe; subapical lobe present on anterior margin. Terminal portion of basimere of T-IX bilobed; L1 modified long extension of apodeme of T-IX paralleling telomere and L2 large rounded lobe bearing two acetabular bristles (ventral short and dorsal long). Telomere narrows from proximal to stiva; stiva expanded dorsoapical angle forming a near right angle. Four long setae on ventral margin of stiva. Fulcral sclerite truncate; very developed. Aedeagus (Fig. 8). Aedeagal apodeme broad, upturned apically. Dorsal margin with thick sclerotization preceding arched median dorsal lobe. Crescent sclerite small, capsule small, satellite sclerite thin, short. Y sclerite reduced. Penis rods thick, short; not reaching end of aedeagal apodeme. Virga ventralis short, thick; half length of aedeagal apodeme. Sclerotized inner tube undulate; prominent dorsal spur at base. Ventral armature absent. Crochet broad at base, abruptly narrowing, scythe-like. Phylax thick, sclerotized. Alpha portion of Ford’s sclerite massive; securifer sharp, hook-like. Tendon of phylax and Ford’s sclerite visible.

**Figure 11. F5:**
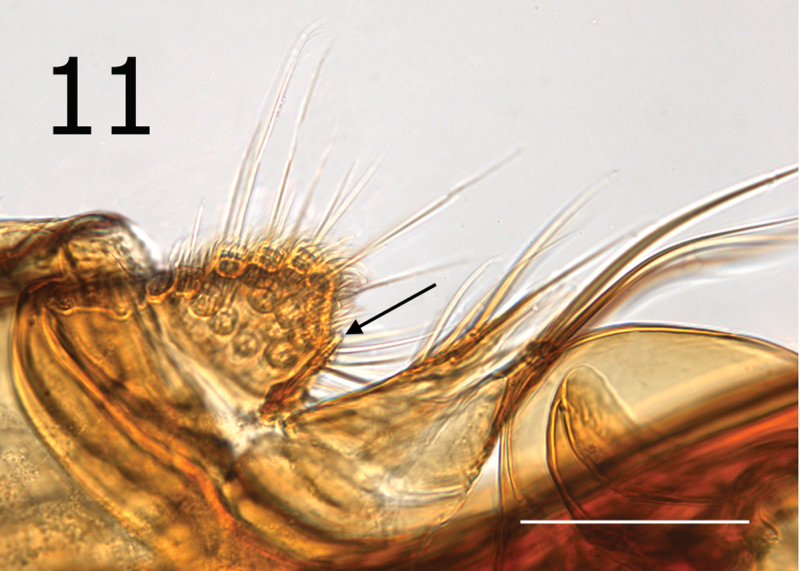
*Lentistivalius philippinensis* sp. n., male holotype (P2316). Sensilium, dorsal and ventral anal lobes, and subsensilial sclerite (arrow). (Scale: 100µ).

**Figures 12–13.**
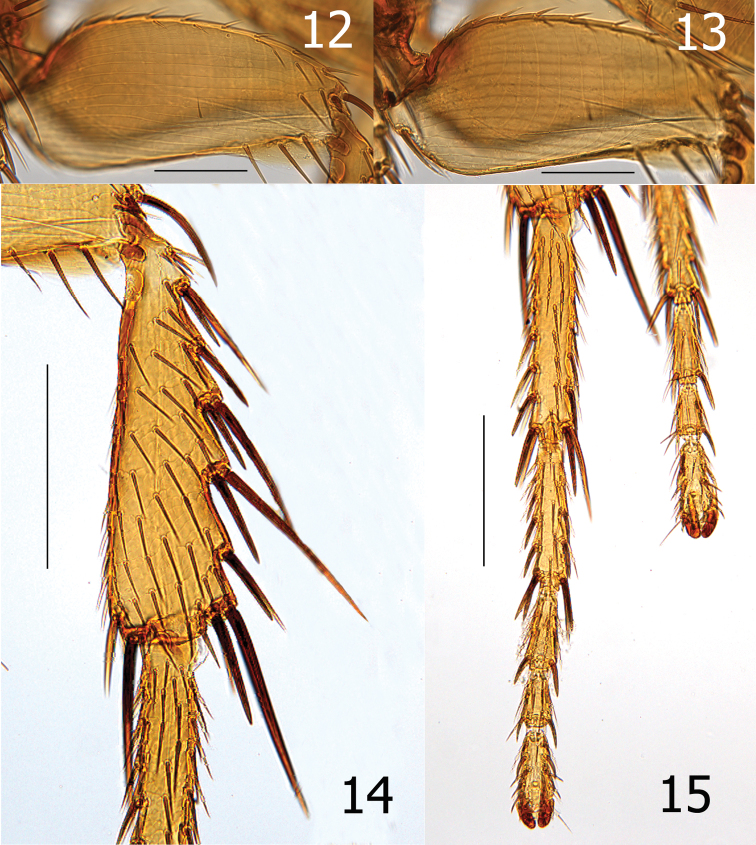
*Lentistivalius philippinensis* sp. n., male paratype (P2211). **12** Lateral of hind femur, longitudinal parallel sculpturing **13** Mesal view of hind femur, vertical parallel sculpturing **14–15** *Lentistivalius philippinensis*, sp. n., male holotype (P2316) **14** Hind tibia **15** Hind tarsi. (Scale: Figs **12–13** = 100µ; Figs **14–15** = 200µ)

##### Etymology.

The new species bears the name of the country from which it was collected.

##### Remarks.

Seven species of *Lentistivalius* are currently recognized (including this new species). *Lentistivalius* is primarily a parasite of Southeast Asian murids and soricids, although one species (*Lentistivalius insolli*) is definitively a bird parasite documented from 18 different species of birds (Hastriter and Bush 2010). A total of 59 *Crocidura grayi* was examined and only two harbored this new species (one flea on each). This flea may occur in greater numbers in months other than May. Additional collecting from *Crocidura grayi* (and other members of Soricidae) at other times of the year (April through August) is needed to discover the undescribed female sex of *Lentistivalius philippinensis* and better define the host and seasonal preferences of this new species.

### Key to species in the Genus *Lentistivalius*

**Table:** 

l	Pronotum with one row of setae	2
1’	Pronotum with two rows of setae (anterior row may only be comprised of one or two small setae dorsally)	7
2(1)	Males	3
2’	Females	5
3(2)	Spiniform setae on ventroapical margin of the distal arm of S-IX appear grouped in a dense patch (India, Nepal, Sri Lanka)	*Lentistivalius ferinus*
3’	Spiniform setae dispersed evenly in a row	4
4(3’)	Ventroapical margin of distal arm of S-IX flat in lateral aspect (Tanzania, Zaire)	*Lentistivalius alienus*
4’	Ventroapical margin convexly rounded (China)	*Lentistivalius occidentayunnanus*
5(2’)	Middle lobe on caudal margin of S-VII large and acutely triangular (China)	*Lentistivalius occidentayunnanus*
5’	Middle lobe smaller and not acutely triangular	6
6(5’)	Caudal margin of S-VII with strongly lobed ventral lobe; several setae on lobe (India, Nepal, Sri Lanka)	*Lentistivalius ferinus*
6’	Lobe weakly indicated; setae clearly not present on weak lobe (Tanzania, Zaire)	*Lentistivalius alienus*
7(1’)	Males	8
7’	Females (females of *Lentistivalius philippinensis* unknown)	11
8(7)	Combination of a substantial spur at the base and dorsal surface of the sclerotized inner tube (s.i.t.) and the width of the distal half of the crochet no wider than the width of the s.i.t. (Luzon, Philippines)	*Lentistivalius philippinensis* sp. n.
8’	Sclerotized inner tube with, or without spur; if spur is present, distal half of crochet is distinctly wider than s.i.t.	9
9(8’)	Stiva of telomere with an angular bulge (near right angle) at dorsoapical angle; not rounded as usual (Japan)	*Lentistivalius aestivalis*
9’	Stiva without angular bulge; evenly rounded	10
10(9’)	Pronotal ctenidia arranged close together and not noticeably reflexed upward towards their apices (Malaysia, Vietnam)	*Lentistivalius insolli*
10’	Pronotal ctenidia separated slightly; diverging towards apices (Borneo)	*Lentistivalius vomerus*
11(7’)	Caudal margin of S-VII without distinct sinus (Malaysia, Vietnam)	*Lentistivalius insolli*
11’	Caudal margin of S-VII with deep sinus (as deep as wide)	12
12(11’)	Undulate dorsal lobe on caudal margin of S-VII with single subtending sinus (Borneo)	*Lentistivalius vomerus*
12’	Dorsal lobe subtended by two sinuses, each separated by a lobe (Japan)	*Lentistivalius aestivalis*

## Supplementary Material

XML Treatment for Ascodipteron speiserianum

XML Treatment for Maabella stomalata

XML Treatment for Thaumapsylla breviceps orientalis

XML Treatment for Thaumapsylla longiforceps

XML Treatment for Ischnopsyllus (Hexactenopsylla) indicus

XML Treatment for Lentistivalius philippinensis
